# A150 ASSOCIATION BETWEEN SERUM USTEKINUMAB CONCENTRATIONS AND ENDOSCOPIC DISEASE ACTIVITY IN CROHN’S DISEASE

**DOI:** 10.1093/jcag/gwab049.149

**Published:** 2022-02-21

**Authors:** R Yanofsky, Y Abduallah, P Golovics, P L Lakatos, A Bitton, G Wild, W Afif, T Bessissow

**Affiliations:** Gastroenterology, McGill University Health Center, Montreal, QC, Canada

## Abstract

**Background:**

Therapeutic drug monitoring, the measurement of serum biologic concentrations and immunogenicity, has become an important method in guiding the management of biologic therapy in patients with Crohn’s disease (CD). Ustekinumab, an inhibitor of the p40 subunit of interleukins 12 and 23, is an approved therapy for patients with CD. However, few studies have explored the relationship between serum ustekinumab drug levels and outcomes in CD. Thus, the utility of serum ustekinumab drug levels in the management of CD remains unknown.

**Aims:**

The primary objective of the study was to evaluate the association between serum ustekinumab drug levels and endoscopic remission (ER) in CD. Secondary outcomes included evaluating the association between serum ustekinumab drug levels and clinical (CR), biochemical, and histological remission (HR).

**Methods:**

Adult patients with CD maintained on ustekinumab were prospectively recruited at the time of routine colonoscopy from 2018 to 2021 at the Montreal University Health Centre, Montreal, Quebec. Clinical and demographic information was obtained from chart and patient review. CD symptom severity was assessed by the Harvey-Bradshaw Index (HBI), with clinical remission defined as an HBI score less than 5. Blood samples were drawn for measurement of serum ustekinumab drug level and C-reactive protein. Stool samples for fecal calprotectin were also collected. Elevated C-reactive protein and fecal calprotectin were defined as a value greater than 5 mg/L and 200 ug/g, respectively. Endoscopic remission was evaluated by the Simplified Endoscopic Score for Crohn’s Disease (SES-CD), with ER defined by an SES-CD score less than 3. If biopsies were taken, histological outcomes were recorded. HR was defined as inactive colitis.

**Results:**

53 patients were included in the study, of which 22 (41.5%) were in ER. Median [interquartile range] ustekinumab drug levels were not associated with ER (ER = 5.4 mg/L [2.6–9.4 mg/L], no ER = 4.3 mg/L [2.3–9.4 mg/L]; P=0.843). There was also no association between quartiles of ustekinumab drug levels and ER (P=0.772). In addition, there was no association observed between median ustekinumab drug level and CR (CR = 4.7 mg/L [2.7–8.3 mg/L], no CR = 3.8 mg/L [2.1–9.6 mg/L]; P=0.993) or HR (HR = 6.4 mg/L [3.5–9.5 mg/L], no HR = 3.7 mg/L [2.2–8.0 mg/L]); P=0.168). There was no association observed between median ustekinumab drug level and C-reactive protein or fecal calprotectin as well (P=0.158 and 0.923, respectively).

**Conclusions:**

There was no association observed between serum ustekinumab drug levels and endoscopic remission. Further studies are required to validate our findings.

**Funding Agencies:**

None

